# Supplementary material for: Ethylene glycol assisted three-dimensional floral evolution of BiFeO3-based nanostructures with effective magneto-electric response
Source: R Soc Open Sci. 2020 Aug 5;7(8):200642. doi: 10.1098/rsos.200642 (PMC7481687; doi:10.1098/rsos.200642)
Supplement: Figure S1 - S4 [file rsos200642supp1.docx]

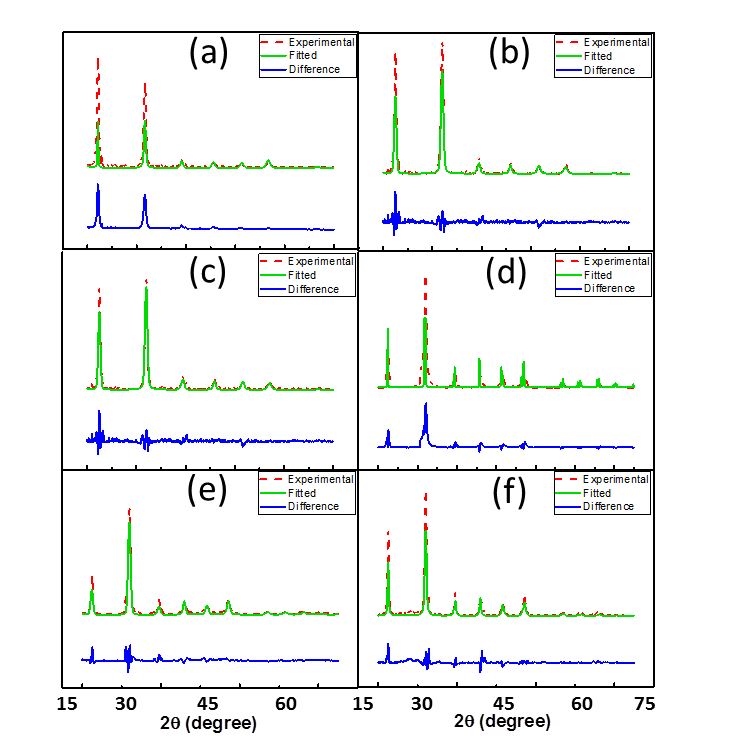


**Fig. S1** Rietveld refined XRD patterns and their corresponding difference plots of the (a) pure BFO, (b) x = 0.00, (c) x = 0.03, (d) x = 0.05, (e) x = 0.07 and (f) x = 0.1 in BiFe_0.9_Co_1-x_Ni_x_O_3_


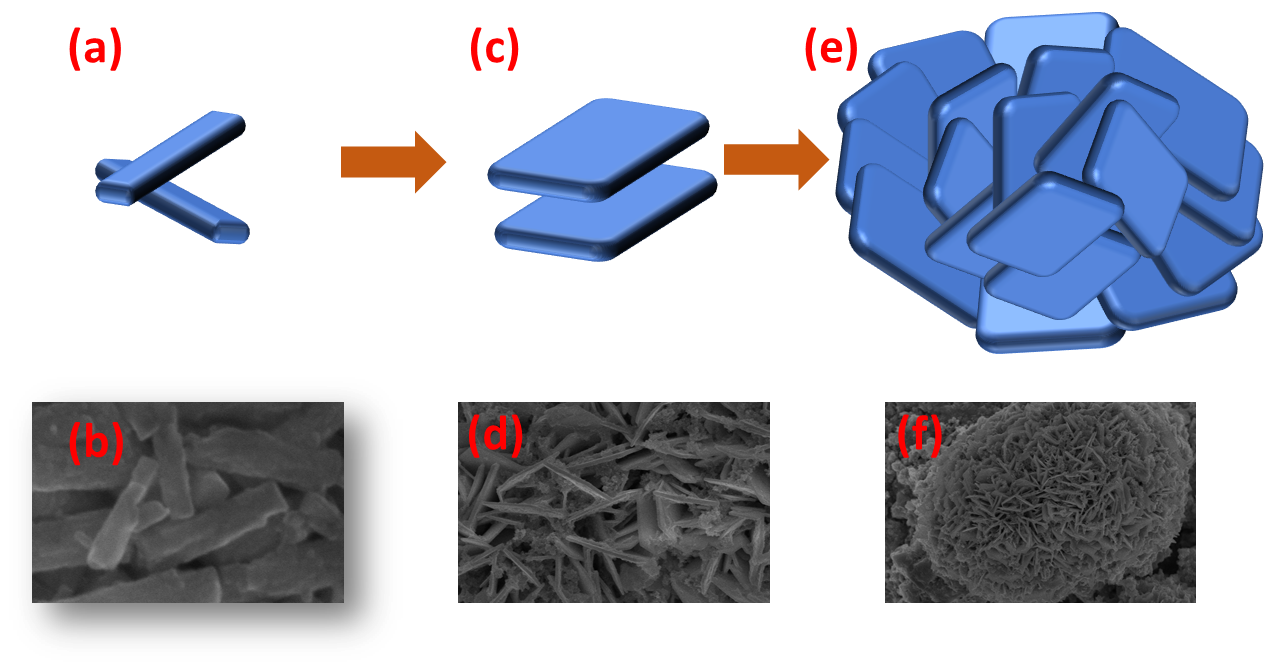


**Fig. S2** Schematic diagram and their real images of (a) & (b) NRs and their transformation to (c) & (d) NPLs and (e) & (f) self –assembled NPLs floral formation, respectively


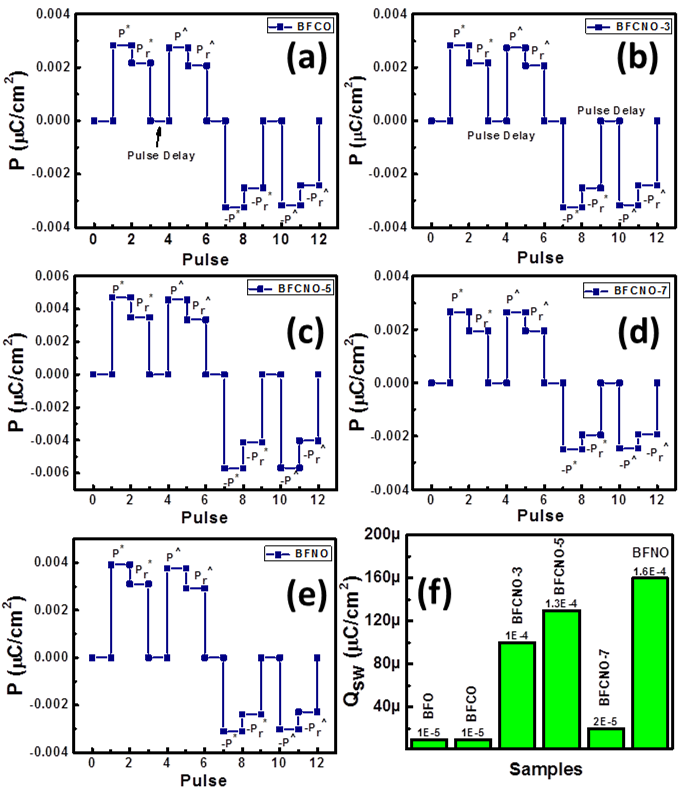


**Fig. S3** PUND sequence for BiFe_0.9_Co_1-x_Ni_x_O_3_ (a) x = 0.00, (b) x = 0.03, (c) x = 0.05, (d) x = 0.07, (e) x = 0.1 and (f) bar graphs of *Q_SW_*


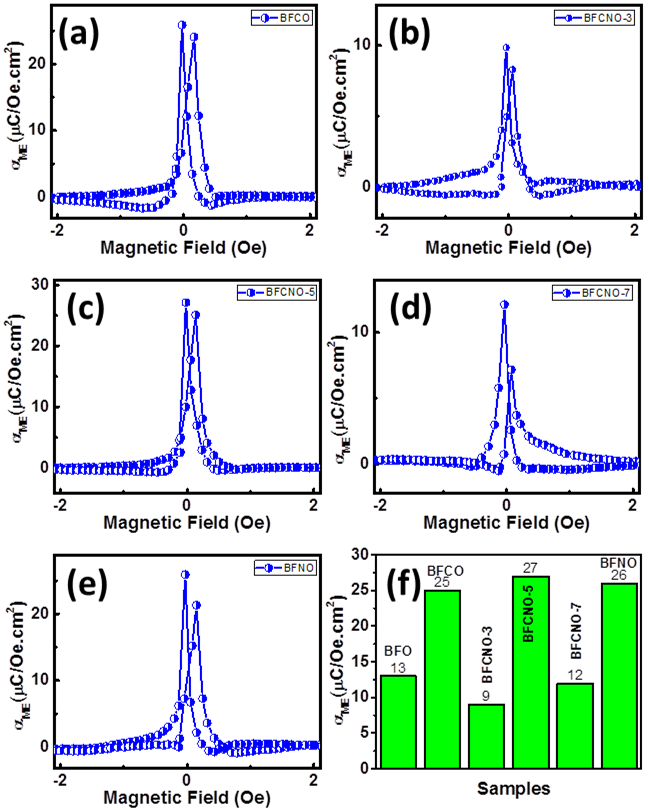


**Fig. S4** α_ME_ vs H plot for BiFe_0.9_Co_1-x_Ni_x_O_3_ (a) x = 0.00, (b) x = 0.03, (c) x = 0.05, (d) x = 0.07, (e) x = 0.1 and (f) bar graphs of α_ME_ for all samples

| **Sample** | **1** | **2** | **3** | **4** | **5** | **6** |
| --- | --- | --- | --- | --- | --- | --- |
| ***a_obs_* (Å)** | 5.591 | 5.592 | 5.595 | 5.586 | 5.593 | 5.599 |
| ***c_obs_* (Å)** | 13.855 | 13.861 | 13.860 | 13.859 | 13.852 | 13.863 |
| ***a_cal_* (Å)** | 5.581 | 5.588 | 5.583 | 5.589 | 5.591 | 5.596 |
| ***c_cal_* (Å)** | 13.799 | 13.867 | 13.829 | 13.773 | 13.795 | 13.830 |
| ***D* (nm)** | 12.2 | 22.4 | 15.1 | 16.2 | 19.5 | 19.6 |
| ***R_exp_*** | 59.41 | 11.34 | 21.01 | 17.12 | 17.32 | 10.12 |
| ***R_weighted_*** | 16.54 | 37.56 | 59.91 | 58.01 | 56.36 | 31.93 |
| ***R_p_*** | 69.48 | 32.28 | 54.79 | 48.98 | 46.23 | 27.67 |
| ***R_bragg_*** | 71.06 | 28.83 | 51.11 | 41.37 | 36.94 | 25.52 |
| ***χ^2^*** | 12.84 | 10.96 | 8.135 | 11.49 | 10.57 | 9.95 |

**Table S1** Lattice constants of observed (*a_obs_* & *c_obs_*) and calculated data (*a_cal_* & *c_cal_*), crystallite size (*D*), and residual parameters including *R-*expected (*R_exp_*), weighted *R*-profile (*R_weighted_*), *R-*profile (*R_p_*), *R*-Bragg (*R*_bragg_), and goodness of fit (χ^2^)

| **Samples** | **BFO** | **BFO-1** | **BFO-2** | **BFO-3** | **BFO-4** | **BFO-5** |
| --- | --- | --- | --- | --- | --- | --- |
| ***P^*^_max_*** | 0.00636 | 0.00169 | 0.00284 | 0.0047 | 0.00266 | 0.00392 |
| ***P^*^_r_*** | 0.00443 | 0.00129 | 0.00217 | 0.0035 | 0.00195 | 0.00311 |
| ***P^^^_max_*** | 0.00635 | 0.00168 | 0.00274 | 0.00457 | 0.00264 | 0.00376 |
| ***P^^^_r_*** | 0.00435 | 0.00127 | 0.00207 | 0.00335 | 0.00195 | 0.00293 |
| ***-P^*^_max_*** | -0.00653 | -0.00175 | -0.00325 | -0.00572 | -0.00250 | -0.00312 |
| ***-P^*^_r_*** | -0.00444 | -0.00135 | -0.00252 | -0.00413 | -0.00196 | -0.00239 |
| ***-P^^^_max_*** | -0.00647 | -0.00174 | -0.00316 | -0.00569 | -0.00246 | -0.00303 |
| ***-P^^^_r_*** | -0.00432 | -0.00134 | -0.00242 | -0.00403 | -0.00193 | -0.00230 |
| ***Q_sw_*** | 0.00001 | 0.00001 | 0.00010 | 0.00013 | 0.00002 | 0.00016 |

**Table S2** Total polarization with switching (***P^*^_max_***), remanance polarization with switching (***P^*^_r_***), total polarization without switching (***P^^^_max_***), remanance polarization with switching (***P^^^_r_***), total polarization with switching in reverse field (-***P^*^_max_***), remanance polarization with switching in reverse field in reverse field (-***P^*^_r_***), total polarization without switching in reverse field (-***P^^^_max_***), remanance polarization with switching in reverse field (-***P^^^_r_***) and switching charge density (***Q_sw_***)
